# 10.E. Round table: Climate emergency, health and equity education: policy and practice recommendations for Europe

**DOI:** 10.1093/eurpub/ckac129.635

**Published:** 2022-10-25

**Authors:** Organised by: ASPHER, Ecorys (Netherlands), EUPHA-ENV, Chair persons: Rana Orhan (ASPHER), Marija Jevtic (EUPHA-ENV)

## Abstract

Climate change continues to be the biggest challenge of the 21st century, with profound and growing negative consequences on public health. While the public health and wider healthcare sector faces the health risks and consequences of climate change, it is also an important actor as producer of carbon footprint. There is therefore a need for public health and healthcare professionals to be prepared to address the climate emergency and its inequitable impacts across vulnerable populations. Climate resilience and climate-health-equity and political literacy should be built to enable public health and healthcare professionals to gain a deeper understanding of the needed changes and communicate the direct and indirect environmental impact of our health system, institutions and daily lives. This round table discussion will be framed by the joint statement “Moving towards the right to ‘health for all’ by training the public health workforce on climate change and health” issued by a network of public health stakeholders under the EU Health Policy Platform. Endorsed by more than 75 organisations from the global, European and national level, the statement calls actors in public health to bring climate change and health concerns to the forefront of the debates. During the round table, 4 panellists will touch on evidence, solutions and guidance to address the climate emergency from the different perspectives of ASPHER, EuroHealthNet, Ecorys and EHESP French School of Public Health. The added value of the round table is conveying the different views of the panellists, bringing together European CSOs and partnerships of public health authorities, academia and a research-based consultancy company, and laying out possible ways of how working together could steer the EU and national multidisciplinary policy and decision-making on the intersection of climate, health, social justice, and education. After setting the need for action, the round table will seek to produce recommendations from public health actors to address the climate emergency, bringing the dialogue parallel to COP27. This workshop will explore how different actors from inside and outside the public health domain can contribute to climate mitigation and adaptation actions. The discussion will cover: investing in climate-health training for public health and healthcare professionals; investing in public health research; designing and monitoring mitigation programmes (e.g., sustainable and resilience-enhancing healthcare policies); building climate-health-equity literacy; and providing space for dialogue for those communities who will be most harmed by an inadequate public health response. From a policy perspective, the workshop will help prepare recommendations for actions related to COP27 and will reflect on strategic processes taking place at the European level (including the Conference on the Future of Europe, the European Health Union, the Strategic Foresight, and the Recovery and Resilience Plans).

**Key messages:**

• Climate change poses an immediate threat to planetary health, and the co-benefits of climate change mitigation and adaptation actions to health and social justice should be highlighted in policies.

• The workshop provides public health actors the opportunity to produce recommended actions from public health actors to address the climate emergency at the European and national level.

**Speakers/Panellists:**

**Tara Chen**

ASPHER, Brussels, Belgium

**Dorota Sienkiewicz**

EuroHealthNet, Brussels, Belgium

**Daniek Korver**

Ecorys NL, Rotterdam, Netherlands

**Laurent Chambaud**

École des Hautes Études en Santé Publique, Rennes, France

